# Lingonberry polyphenols: Potential SARS‐CoV‐2 inhibitors as nutraceutical tools?

**DOI:** 10.14814/phy2.14741

**Published:** 2021-02-01

**Authors:** Pirjo Pärnänen, Hanna Lähteenmäki, Ismo Räisänen, Taina Tervahartiala, Timo Sorsa

**Affiliations:** ^1^ Faculty of Medicine Department of Oral and Maxillofacial Diseases Head and Neck Center University of Helsinki and Helsinki University Hospital Finland; ^2^ Department of Dental Medicine Karolinska Institutet Huddinge Sweden

## Abstract

Proposed pathway of the effect of lingonberry polyphenols on oral microbial (viral) load reduction and consequent beneficial local and systemic (respiratory tract) anti‐inflammatory and antimicrobial/antiviral effects.

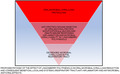

Dear Editor,

We have read with great interest the article “Mucin signature as a potential tool to predict susceptibility to COVID‐19” by Bose et al. (2021) on mucins and their glycosylation alterations in the upper respiratory tract and their role in innate immunity and infectious disease progression. With this keeping in mind, oral mucus has a distinct set of mucins (Frenkel, & Ribbeck, 2015) which are involved in the defense against pathogens capable of degrading or modifying proteins of the epithelial layer and saliva.

It has been proposed by Sampson et al. (2020) that oral bacterial burden and oral health may affect the outcome of COVID‐19 complications. If oral immune responses fail, the oral cavity acts as a passway for pathogens further to the lungs.

There is substantial in vitro evidence that polyphenols (synthesized or from natural sources) act as antiviral agents against viruses, such as SARS‐CoV, MERS‐CoV, MERS/SARS‐CoV, and their specific cysteine proteases, Epstein–Barr virus, enterovirus, Herpes simplex, influenza A virus, Coxsackievirus B1, West Nile, Zika, and Dengue viruses. The active molecules identified were: (−)‐catechin gallate and (−)‐gallocatechin gallate, resveratrol and its analogs, synthetic curcumin, polyphenols, delphinidin, quercetin and fisetin, and anthocyanins.

The inhibitory mechanisms of polyphenols against viruses vary depending on their composition: they include dose‐dependent viral load or nucleocapsid protein expression reduction, inhibition of viral binding activity and replication, inhibition of viral protease and enzyme activities, alteration of the viral structure or membrane vesicles, stimulating host's immune system by blocking viral surface glycoproteins or by reducing interleukin levels generating the anti‐ inflammatory outcome. Hydroxyl groups in polyphenols play eventually important roles in these activities. A key mechanism for COVID‐19 is the upregulation of the MAPK p38 pathway, which plays a crucial role in the upregulation of proinflammatory cytokines such as interleukin‐6 (IL‐6), tumor necrosis factor‐α (TNF‐α), and IL‐1β, and loss of angiotensin‐converting enzyme activity causing damage in the lungs and heart (Grimes & Grimes, 2020). It has been, indeed, proposed by Annunziata et al. (2020) that polyphenols may have a role against coronavirus infection, and that polyphenolic extracts, rather than isolated polyphenols, may ensure these multiple actions.

Lingonberries (*Vaccinium vitis idaea* L.) have a unique phenolic composition of which at least 28 have been identified including flavonoids (anthocyanins, flavonols [e.g., quercetin], flavanols [catechins]), phenolic acids, lignans, stilbenes (resveratrol), and phenolics polymers (e.g., proanthocyanidins) of which anthocyanins, flavonols, and proanthocyanidines are the main constituents. Resveratrol may be extracted from berry peels and seeds. Phenolic compounds of lingonberries are absorbed through the intestines and retain their biological activity in the ileal fluid samples. In in vivo studies, the main anthocyanin of lingonberries cyanidin‐3‐O‐galactoside and its methylated/ glucuronidated metabolites were excreted in urine or feces. Lingonberries do not markedly change the permeability of high permeable drugs in vitro.

Lingonberry cyanidin has been shown to inhibit in vitro p38MAPK phosphorylation, compared to blueberry extract, which also inhibits the phosphorylation of JNK in retinal photoreceptor cells after UV exposure (Ogawa et al., 2013). Lingonberry fruit extract has been shown in vitro to downregulate inflammatory mediators such as IL‐6, TNF‐α, IL‐1β, Monocyte chemotactic protein 1, cyclooxygenase‐2, and inducible nitric oxide synthase reducing mouse adipocyte inflammation (Kowalska et al., 2019).

Lingonberries have also been shown to retain their beneficial in vitro and in vivo antimicrobial (e.g., *Candida*, *S. mutans*, periodontopathogens) and anti‐inflammatory properties in the form of fermented lingonberry juice in a clinical human study (Pärnänen et al., 2019). This study evaluates natural‐based polyphenols, especially derived from lingonberries, as plausible antiviral, and anti‐inflammatory agents and this effect could be monitored with an oral rinse point‐of‐care test. Fermented lingonberry juice is a safe, natural product and may be used locally as a mouthwash (or may be swallowed to obtain systemic effects) to reduce the oral microbial load and subsequent low‐grade inflammation caused by host inflammatory cytokines, for example, IL‐6, TNF‐α, and by microbial actions. It is specially designed to contain low sugar content for safe and effective oral use.

Could it be that there are variations in innate immune response to pathogens in the oral cavity caused by microbial burden, altered individual oral mucin‐ architecture and subsequent infection spread into the lungs and could these events be prevented by polyphenols from lingonberry? Nutritional aspects regarding COVID‐19 and future pandemics have been brought to attention by Jaggers et al. (2020) and Zabetakis et al. (2020). Severe inflammatory reactions are detected in COVID‐19 patients and our hypothesis is that polyphenols as a nutritional tool could inhibit viral actions by multiple mechanisms, alleviate inflammatory reactions early on in disease development, and could prevent the escalation of the inflammatory processes aiding in recovery from COVID‐19 and should be studied keeping in mind forthcoming pandemics.

## CONFLICT OF INTERESTS

Dr Pirjo Pärnänen is the inventor of patent EP 2585087B1, and Professor Timo Sorsa is the inventor of US patent 10488415B2 and Japan patent 2016‐554676.

## DATA AVAILABILITY STATEMENT

The data that support these findings are available upon reasonable request from the corresponding author.
